# Anatomopathological uterine findings of Candida albicans infection in a vulvovaginal model

**DOI:** 10.1099/jmm.0.002160

**Published:** 2026-05-07

**Authors:** Valéria Mosca, Glaucia Sayuri Arita, Karina Mayumi Sakita, Juliana Aparecida Fernandes, Rita de Cássia Lima Ribeiro, Francieli Gesleine Capote-Bonato, Denis Vinicius Bonato, Marcelo Marcondes Seneda, Erika Seki Kioshima, Patricia de Souza Bonfim-Mendonça, Terezinha Inez Estivalet Svidzinski

**Affiliations:** 1 Medical Mycology Laboratory, Department of Clinical Analysis and Biomedicine, State University of Maringá, Maringá, Brazil; 2 Department of Veterinary Clinics, State University of Londrina, Londrina, Brazil; 3 Department of Animal Sciences, Paranaense University (UNIPAR), Umuarama, Brazil

**Keywords:** candidiasis, experimental model, female reproductive system, uterus

## Abstract

**Introduction.** Vulvovaginal candidiasis (VVC) is a common fungal infection that has a significant impact on global public health. Although studies associate VVC with male infertility, its influence on the female reproductive system, particularly uterine involvement, remains unknown.

**Gap statement.** While recent animal studies propose that *Candida albicans* migration from the vaginal tract to the uterus in VVC could lead to infertility, the underlying histopathological alterations that support this connection are not well understood.

**Aim.** To investigate possible changes in the uterine tissue of BALB/c mice infected experimentally with *C. albicans*, by analysing the progression and effects of infection on the uterus.

**Methodology.** Female BALB/c mice were divided into two groups (infected and control). Vaginal infection was induced by *C. albicans*, and vaginal and uterine tissues were collected at different intervals (1, 3, 5, 7 and 10 days). Analyses included fungal burden (c.f.u. g^−1^), histopathology stained with Grocott–Gomori and macroscopic and microscopic evaluation (haematoxylin–eosin staining) of uterine tissue.

**Results.** Vaginal infection was confirmed by a consistent presence of yeast in vaginal tissue. *C. albicans* migration was observed in the uterus, with a significant increase in fungal burden on day 3, followed by macroscopic alterations such as oedema and hyperaemia. Histologically, inflammatory infiltrates, epithelial necrosis and progressive degeneration were identified until day 7, with signs of resolution by day 10.

**Conclusion.** The results demonstrate that vaginal infection by *C. albicans* was able to cause significant uterine alterations with self-limiting progression. These findings suggest that VVC may have direct implications for female fertility, warranting future investigations into its influence on infertility cases.

## Introduction

Vulvovaginal candidiasis (VVC) is a public health problem with worldwide impact, affecting women across all social classes. Several factors are associated with the emergence of VVC, including genetic predispositions such as blood group polymorphisms, hormonal changes, antibiotic use, age, sexual activity, diseases such as diabetes mellitus and other unclear causes that can contribute to colonization by *Candida* spp. or infection (VVC) development [1].

VVC can be classified as complicated or uncomplicated. Uncomplicated cases correspond to mild and sporadic infections, mainly caused by *Candida albicans* [2]. Conversely, complicated cases involve severe episodes, often attributed to other *Candida* species or specific situations such as VVC during pregnancy or when affecting immunocompromised or diabetic women [3]. Particular cases like recurrent VVC (RVVC), characterized by four or more episodes within a year, are also considered a complicated infection [4]. Despite this classification, the consequences of VVC in any scenario remain poorly documented. It is unclear what occurs within the uterus, even in simple cases, that could justify complications stemming from vaginal infection.

Experimental models have shown an association between VVC and infertility [5]. It is rational to consider that the proximity of female genital organs could be a risk factor for complications arising from vaginal tissue infection. However, a gap exists in the literature regarding detailed recognition of uterine involvement by yeast following vaginal infection. Recently, our research group has proved that *C. albicans* can migrate from the vagina to the uterus after a vaginal infection [6]. However, the actual involvement and impairment of the uterus are still unclear.

Furthermore, the prevalence of VVC in pregnant women is higher than in non-pregnant women [7], but the real consequences remain inconclusive [5]. The impact of *Candida* infection on male infertility is well-documented, as studies have observed reduced motility and survival of spermatozoa exposed to *Candida* yeast [8,11]. Additionally, recent research reported DNA alterations in sperm exposed to *Candida* sp., as well as necrosis and apoptosis, suggesting an overall compromised sperm quality [12,14]. These findings support the idea that *Candida* may directly affect male fertility. On the other hand, the effects of this infection on female fertility are not well elucidated. Although VVC is frequently observed in women with infertility disorders, the association between altered vaginal microbiota and infertility remains unclear. Some authors suggest a relationship between fungal infection and infertility, but the evidence remains inconclusive, with few available corroborative studies [15,16]. Moreover, according to Córdova *et al.* [8], no significant difference has been found between fertile and infertile women concerning candidiasis. These authors suggest that VVC alone may not be directly related to infertility. This data indicate that despite hypotheses about fungal infections influencing female fertility, current evidence is insufficient for robust confirmation.

This debate is highly relevant; however, little is proven about possible uterine involvement by yeast due to VVC. Recently, our research group demonstrated that yeast inoculated into the vaginal cavity of mice rapidly migrated to the uterus of experimentally infected animals [6]. However, it remains unclear whether it occurred as a mere colonization or an intrauterine invasive fungal infection. Thus, this study aimed to analyse possible uterine morphological and histopathological changes that could be attributed to yeast ascending from the vaginal lumen of mice infected experimentally with *C. albicans*.

## Methods

### Animals and ethical statement

This research was approved by the Institutional Ethics Committee for Animal Experimentation at the State University of Maringá, Brazil (CEUA No. 2253200623). Female BALB/c mice, ~6 weeks old (weighing 21–23 g), were housed under standard conditions (temperature 20–22 °C, a 12 h light/dark cycle and ad libitum feeding) at the State University of Maringá, Brazil. The animals were handled and treated according to the US National Institutes of Health (NIH) Guide for the Care and Use of Laboratory Animals guidelines.

### Experimental design

In total, 50 mice were randomly divided into two groups: (1) uninfected (*N*=25) and (2) experimentally infected with a clinical isolate of *C. albicans* (*N*=25). Vaginal infection procedures were conducted on two different days (totalizing one hundred animals). All mice received a subcutaneous injection of 0.1 ml of *β*-estradiol 17-valerate (Sigma, USA) dissolved in sesame oil, equivalent to a dose of 0.3 mg per animal, administered periodically throughout the experiment. For infection, suspensions of *C. albicans* (1×10^6^ yeast ml^−1^) in 30 µl of PBS, pH 7.4, were inoculated within the vaginal lumen 48 h after the second oestrogenization. Animals from the uninfected group received just PBS (30 µl). Five mice from each group were euthanized using isoflurane (Isoforine, Cristália, Itapira – São Paulo, Brazil) on days 1, 3, 6, 7 and 10 post-infection. Vaginal and uterine tissues were collected for fungal burden assessment and histopathological analyses. Longitudinal sections of the vagina and uterus were prepared, with the right half of each organ designated for fungal burden quantification and the left half for histopathological evaluation.

### *Candida* strain

A clinical isolate of *C. albicans* obtained from a previous research approved by the Committee for Ethics in Research with Humans – COPEP, at UEM (report no. 435/2009) was used for the infection. This yeast was isolated from a woman with VVC, and it has been preserved at −80 °C in the Laboratory of Medical Mycology at the State University of Maringá, Brazil.

### Fungal burden

Vaginal and uterine tissues were excised, and half of each organ (right side) was weighed and macerated in sterile ceramic crucibles using pistils along with 1,000 µl of lysis buffer (200 mM NaCl, 5 mM EDTA, 10 mM Tris, 10% glycerol v/v, pH 8.3). The suspensions of macerated tissues were plated onto Sabouraud dextrose agar culture medium (Kasvi, Laboratories Conda S.A., Spain) and incubated at 35 °C for 24 h to determine the fungal burden expressed as c.f.u. per gram of tissue (c.f.u. g^−1^).

### Histopathological analysis

Part of the vagina and uterus tissues (left side) were fixed in 4% paraformaldehyde for 24 h, preserved in 70% ethanol, and then embedded in paraffin. The organs were subsequently cut longitudinally into 5 µm sections. To evaluate cell characterization and tissue response to *C. albicans* infection, slides were analysed across 100 fields in at least six histological sections. Cellular structures were observed and photographed using a binocular light microscope attached to a camera (Motic BA310 – Moticam 5 camera) at 400×magnification. For fungal visualization, some sections were stained with Grocott–Gomori methenamine silver (GMS) and counterstained with haematoxylin and eosin (H and E).

### Statistical analysis

The data distribution was verified using the Kolmogorov–Smirnov. Statistical analyses were conducted using one-way ANOVA with a Bonferroni post-test, using GraphPad Prism version 8.0.2. *P* values <0.05 were considered statistically significant. The experiments were carried out in two independent assays.

## Results

Firstly, the vaginal environment of mice was prepared with oestrogen injection, aiming for a favourable condition for infection and development of VVC.

### Assessment of fungal burden in experimental VVC

**Fig. 1.**
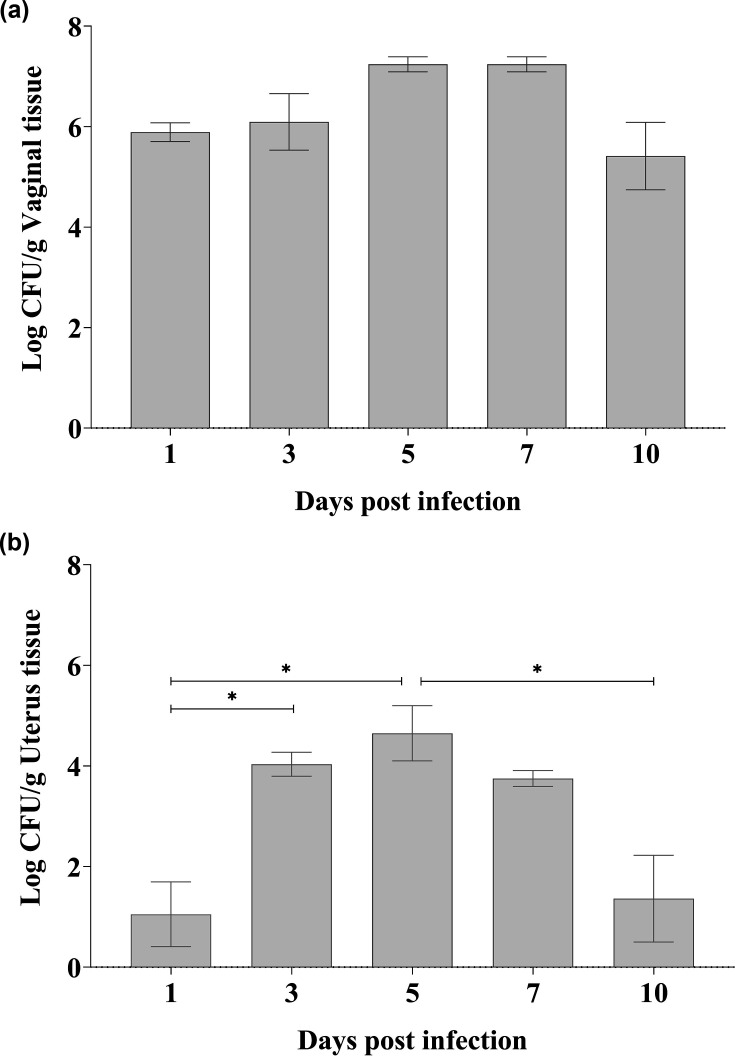
Fungal burden in (a) vaginal and (b) uterine tissues following VVC infection. *P<0.05, error bars represent sd, *n*=5 animals/day infection. Experiments performed in duplicate.

To confirm the infectious process, the colony count was estimated, expressed as c.f.u. g^−1^ of vaginal tissue, enabling comparative analysis with previous studies conducted by the group. Fig. 1 shows the experimental success of the infectious process in the vagina of BALB/c mice and the fungal burden in the uterus. In vaginal tissue, the fungal burden remained stable throughout the experiment (without statistical difference) (Fig. 1a). This result suggests a robust and well-consolidated VVC process. Regarding the fungal burden in the uterus, the analysis revealed that the growth of *C. albicans* was low on the first day post-infection. However, a considerable count, nearly four logs, with a statistically significant increase was observed, until the fifth day, followed by a gradual decline in these values until the tenth day (Fig. 1b).

### Yeasts inside the uterine tissue

**Fig. 2.**
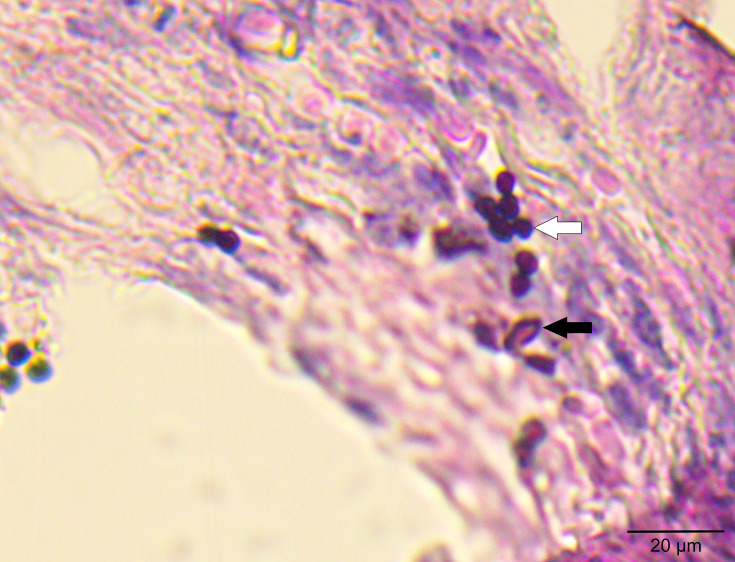
Histological sections of uterine tissue stained with GMS and counterstained with haematoxylin–eosin (H and E). Yeasts (white arrow) and hyphal fragments (black arrow) observed by light microscope (Motic BA310 – Moticam 5 camera) at 400×magnification.

Reinforcing the fungal load data, Fig. 2 shows that the yeasts inoculated into the animal’s vaginal lumen were able to reach the uterine tissue. It was possible to observe the presence of both single and grouped yeast cells along with hyphal fragments, suggesting an invasion process of *C. albicans* in the uterine tissue during experimental VVC.

### Uterus: macroscopic aspects

**Fig. 3.**
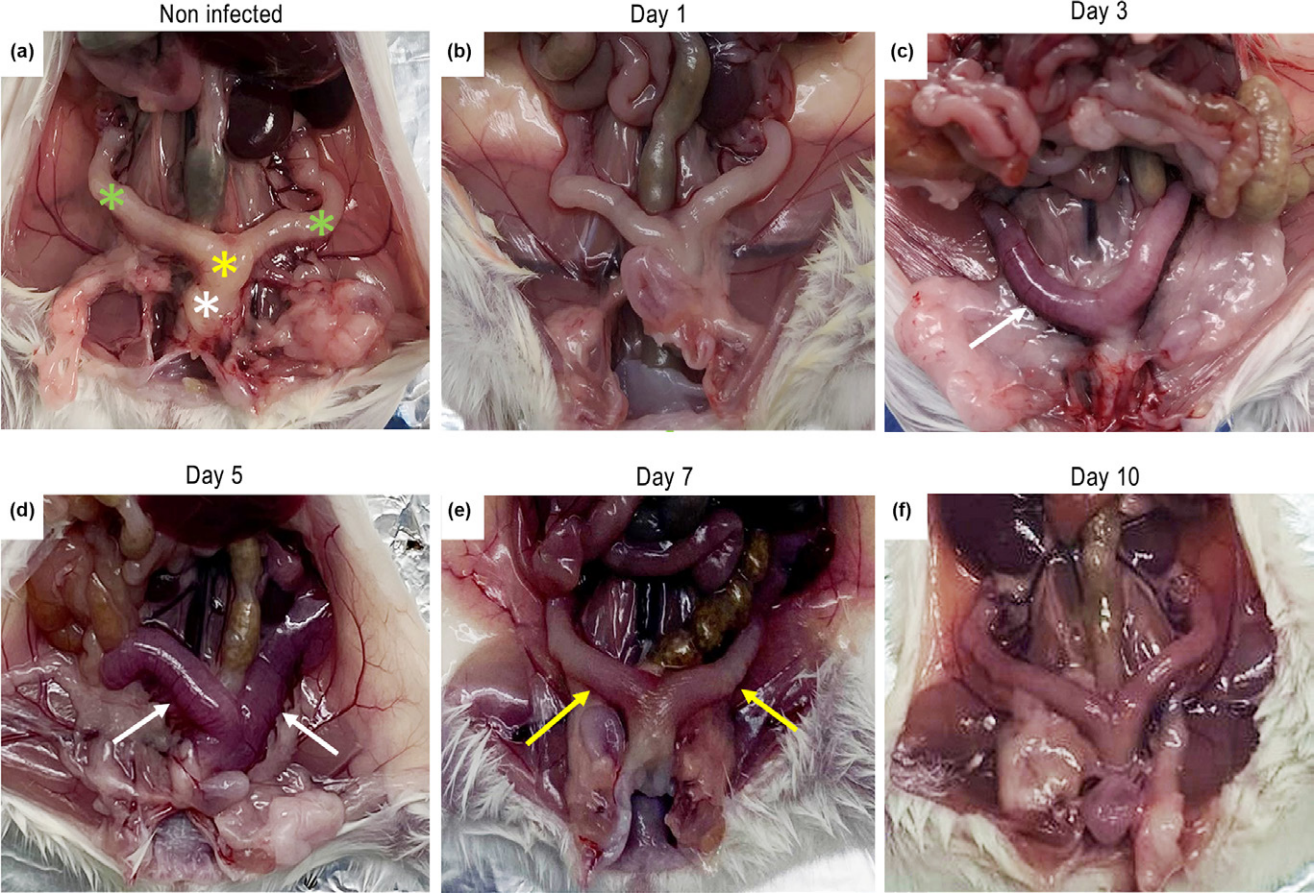
Macroscopic aspects of uterine morphology: (a) non-infected animal from control group and (b)–(f) post-infection of mice intravaginally infected with *C. albicans*. Asterisks indicate the main mice uterus structures: uterine body (yellow), cervix (white) and the right and left horns (green). Arrows indicate clinical changes: serosal oedema with diffuse hyperaemia (white), pronounced diffuse hyperaemia over both uterine horns and the uterine body (yellow), highlighting the serosa that has acquired a uniform dark colour with a granular tissue appearance (**f**).

The animals that were experimentally infected intravaginally with *C. albicans* exhibited signs compatible with an infectious process, such as increased uterine volume, oedema and intensified vascularization (Fig. 3). The topography of the uterus in non-infected BALB/c mice (Fig. 3a) is located in the caudal portion of the abdominal cavity and adhered to the dorsal wall of the abdomen. The uterus is formed by a central tubular part called the body of the uterus (yellow asterisk), located cranial to the urinary bladder and covered by a serosa adhered to the abdominal wall. Its most caudal portion is called the cervix (white asterisk). In addition to the body, the uterus consists of two tubular structures, right and left, known as horns (green asterisks). The next images macroscopically illustrate the progression of VVC infection on uterine changes over the evaluated days. On day 1, slight serosal oedema is observed in both right and left uterine horns and the uterine body (Fig. 3b). The infectious process becomes more evident from day 3, when an increase in the organ’s volume as a whole, moderate serosal oedema, and pronounced diffuse hyperaemia in the right uterine horn (arrow, Fig. 3c) are visible. On day 5, the alterations were accentuated, and hyperaemia can be observed in both uterine horns, which appear with thin walls and evident oedema (arrows). From day 7, the process seems to stabilize naturally, with still pronounced diffuse hyperaemia over both uterine horns and the uterine body; the serosa has acquired a uniform dark colour, resembling a granular tissue appearance (yellow arrow, Fig. 3e). On day 10, only slight and focal hyperaemia is restricted to the serosa of the uterine horns. To summarize, notably, on days 3 and 5, the most significant macroscopic alterations were observed, including pronounced oedema and a marked increase in vascularization. These results are consistent with Fig. 1(b), which shows a statistically significant difference in the fungal burden of uterine tissue on days 3 and 5 post-infection.

### Uterus: histopathological aspects

**Fig. 4.**
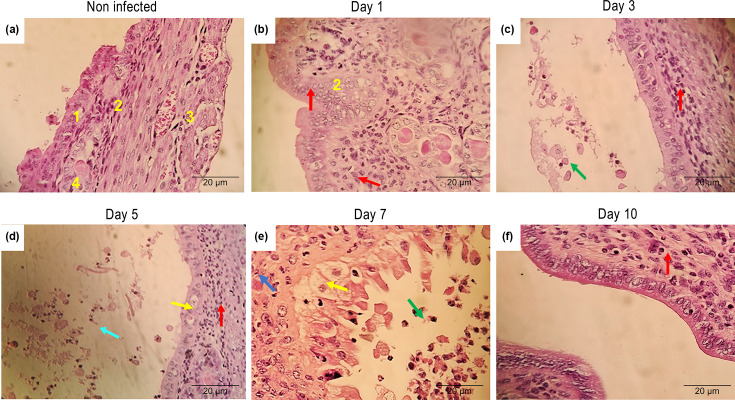
Histopathological findings in the uterine epithelial tissue of BALB/c mice infected intravaginally with *C. albicans*. The mucosal layer of the endometrium [1], the submucosal layer of the endometrium [2], the myometrium [3] and the uterine glands [4]. The arrows indicate amorphous and purulent material with the presence of neutrophils (red), macrophages (green), purulent material (brown) and pronounced and diffuse degeneration and necrosis of the epithelial layer of the mucosa (yellow). All images were captured at 400×magnification.

From a histopathological point of view, the uterine lumen of a normal mouse is composed of four main structures: the mucosal layer of the endometrium [1], the submucosal layer of the endometrium [2], the myometrium [3] and the uterine glands [4], represented in Fig. 4(a). One day after infection, the submucosal layer of the endometrium already shows an inflammatory infiltrate composed of neutrophils, identified by the red arrow (Fig. 4b). Three days post-infection, the persistence of amorphous and purulent material in the uterine lumen, containing neutrophils (red arrow) and macrophages (green arrow), was identified. The submucosal layer of the endometrium maintains the inflammatory infiltrate of neutrophils (red arrow), suggesting an ongoing inflammatory process (Fig. 4c). On day 5 post-infection, the uterine lumen exhibits amorphous content, composed of neutrophils (red arrow), macrophages and activated macrophages (green arrow), as well as purulent material (brown arrow). There is also a slight and focal degeneration of the epithelial layer of the mucosa (yellow arrow), accompanied by a neutrophilic inflammatory infiltrate in the submucosal layer (red arrow) (Fig. 4d). On day 7 post-infection, the submucosal layer still presents a neutrophilic inflammatory infiltrate (red arrow). There is marked and diffuse degeneration and necrosis of the epithelial layer of the mucosa (yellow arrow), indicating progressive tissue damage and persistence of the luminal content composed of neutrophils and macrophages (green arrow) in Fig. 4(e). Finally, on day 10 post-infection, only a slight neutrophilic inflammatory infiltrate was observed in the submucosal layer (red arrow), with the uterine lumen showing no evident content, suggesting a possible partial resolution of the inflammatory process (Fig. 4f).

### Uterine tissue: histopathological details

**Fig. 5.**
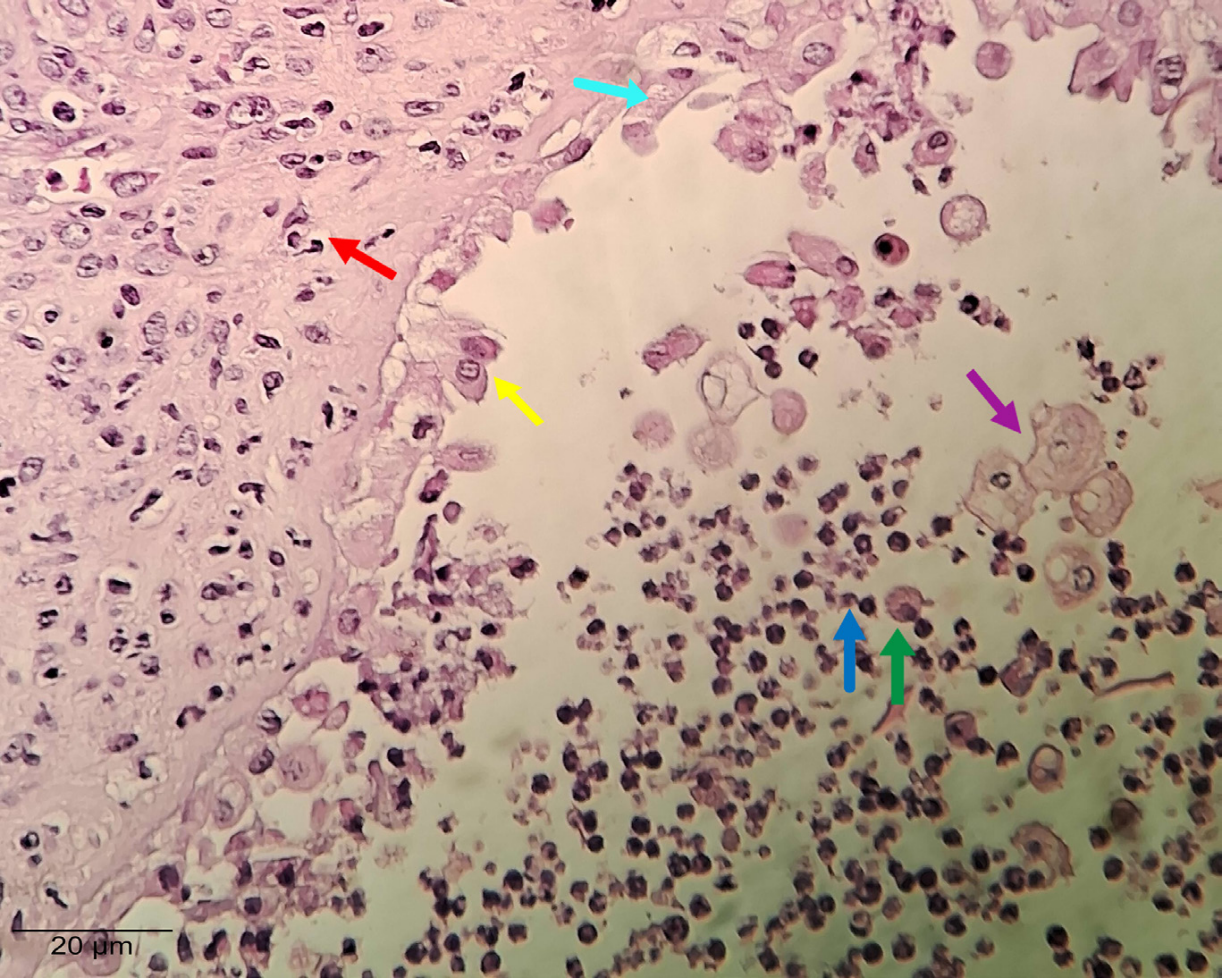
The peak of the inflammatory process in the uterus (day 7 post-infection): The arrows indicate neutrophils (blue), activated macrophages (green), significant diffuse alterations of the epithelial layer of the mucosa (yellow) and desquamation into the uterine lumen (black). All images were captured at 400×magnification.

Fig. 5 details the main histopathological changes observed in the uterine tissue of the animals experimentally infected intravaginally with *C. albicans*. After 7 days of infection (the peak of the inflammatory process in the uterus), the presence of purulent luminal content is noted, highlighting neutrophils (blue arrow) and activated macrophages (green arrow). At this stage, there is pronounced degeneration and necrosis, with significant diffuse alterations of the epithelial layer of the mucosa (yellow arrow). It is observed a loss of adhesion of the epithelial cells and desquamation into the uterine lumen (black arrow). In the submucosal layer, the presence of a neutrophilic inflammatory infiltrate is noted (red arrow).

## Discussion

Our research group has been studying VVC for several years; recent investigations show that after experimental VVC, yeasts can migrate from the vaginal canal to the uterus [6]. In this context, the hypothesis is that the presence of yeast in uterine tissue results in pathological changes and organ dysfunction. In fact, there is limited information about VVC causing uterine epithelial alterations and pathological consequences such as infertility.

In this work, initially, the murine vaginal VVC model was validated through the evaluation of fungal burden in the vaginal tissue (Fig. 1a). The presence of the yeasts that had been inoculated into the lumen was detected in cultures of the macerated vaginal tissue since the first day evaluated. This finding suggests that the invasion process occurred, as already established in previous studies from our research group [6,17,19] and from other authors [20]. In addition, quantification of fungal burden in the uterine tissue confirms the migration of yeast from the vagina to the uterine tissue. Infection kinetics showed a consistent infection in the uterine tissue from day 3, which persisted until day 7, when the infection was evaluated (Fig. 1b). Fig. 2 shows histological sections of uterine tissue; the presence of yeasts and hyphae can be seen, confirming the infectious process. The yeast form (blastoconidia) generally suggests colonization, which may or may not induce infection and defence response. On the other hand, the presence of hyphae, an important virulence attribute of *C. albicans*, is strongly associated with tissue invasion and cellular damage [21]. These events are recognized by the host, provoking an immune response. This morphological transition in *C. albicans* is a key event for triggering cellular changes and the development of subsequent immunopathological processes [21]. The migration of yeasts into the uterus has already been inferred in clinical studies. Four cases of intrauterine candidiasis associated with clinicopathological features were reported [22] and drew attention to prematurity and death. In addition, some literature reviews showed several cases of congenital systemic candidiasis [23,24]. There are two recognized routes for establishing the foetal infection: the first one is based on the presence of *Candida* in the maternal circulation, which can reach the foetal circulation via the placenta. The second one is highlighted by the authors as an ascending route, the most likely, where *Candida* migrates from the maternal genital tract through ruptured or intact foetal membranes and infects the amniotic fluid [23]. This context prompted us to deepen our experimental knowledge of the changes in uterine tissue after VVC.

The presence of *C. albicans* within the uterine tissue was observed, along with macroscopic changes, such as oedema and hyperaemia of the organ (Fig. 3), typical of an infectious process [25]. These authors reported a significant increase in uterine volume, which presented thin walls and fluid accumulation, conditions also compatible with the development of *Candida* sp. External clinical (macroscopic) aspects of the uterus were monitored and described as a consequence of the experimental vaginal infection over 10 days (Fig. 3). A joint analysis of Figs 1 and 3 shows that the yeasts quickly reached the uterine tissue, with an expressive colony count on day 3. Moreover, there was coherence between this finding and the macroscopic manifestation of the uterus, which exhibited changes such as increased volume and pronounced diffuse hyperaemia affecting both horns. This progression of signs occurred from day 3 to day 7. In this regard, our study shows for the first time histopathological monitoring of the uterus after experimental VVC. As shown in Fig. 4, initially, a discrete and focal inflammatory process was evident on days 1 and 3. By day 5 (the highest c.f.u. number of the entire process), it was noted that the inflammation reached the other layers of the uterus, which is in agreement with the macroscopic images (Fig. 3). The peak of the inflammatory and degenerative process occurred on day 7 (Fig. 5), when, in addition to this diffuse inflammatory process, there was detachment of the endometrial layer, which was degenerated and necrotic, reflecting the decrease in colony count. Changes compatible with those presented in Figs 4 and 5, such as necrosis, infiltrate and hyperaemia, were found in the uterine tissue of pregnant rats experimentally infected with *C. albicans* [26]. These authors also recorded endometrial fibrosis attributed to the strong inflammatory response. Desquamation, oedema and necrosis of the epithelial mucosa of the endometrium were also reported in those animals, suggesting a significant inflammatory process. Histological changes showed submucosal hyperaemia and destruction of epithelial tissue, compatible with the pathogenic effect of the infection and inflammatory infiltration composed of neutrophils and lymphocytes [27]. It is unquestionable that in the present study, there was a uterine infection, which developed as a consequence of intravaginal infection, confirmed by the data obtained from the culture of uterine tissue (Fig. 1).

Studies showing uterine involvement during experimental VVC are scarce. Chen and Kong [28] reported similar results, such as uterine oedema, associated with high infection levels. They also found submucosal hyperaemia and an inflammatory process, ulceration and infiltration of neutrophils and lymphocytes in the submucosal tissue. Mosci *et al.* [25] reported an intense inflammatory response in the uterus of mice, including necrosis of the mucosal epithelium and neutrophil infiltration.Consistent results with the present study were found, but they were evaluated with a different focus, which prevented a more in-depth comparison with our results.

However, despite the evident involvement of the uterus, the relationship between *Candida* infections and infertility has not yet been fully proven. Vander and Prabha [5] expanded knowledge on this subject, but the authors performed repeated infections (re-infecting the animals every 10 days of experimentation). The authors stated that the presence of *Candida* in the vaginal environment caused infertility after mating. Some histopathological alterations observed in the current study seem to support this hypothesis. However, our results do not allow this conclusion since the vaginal infectious process was induced a single time. Despite that, it is possible to infer that in cases of multiple reinfections, the uterine alterations might not have been self-limiting, which will likely impact the fertilization. This reflection is quite relevant as it could be extrapolated in cases of women with RVVC, who might experience the same uterine complications found in this study and consequently could infer a correlation with infertility.

To conclude, this study presents for the first time a complete investigation of the uterine events after experimental vaginal infection with *C. albicans*. The consistent fungal burden for more than 5 days in the uterine tissue, associated with significant histopathological alterations, provides the first indications towards understanding the possibility of VVC playing a leading or supportive role in infertility processes. However, further studies are needed to better understand how impairment of the female reproductive system, especially in women with RVVC, can impact infertility.

## Abbreviations

GMS: Grocott–Gomori methenamine silver
NIH: National Institutes of Health
RVVC: recurrent VVC
VVC: vulvovaginal candidiasis

## References

[1] Gonçalves B, Ferreira C, Alves CT, Henriques M, Azeredo J (2016). Vulvovaginal candidiasis: epidemiology, microbiology and risk factors. Crit Rev Microbiol.

[2] Sobel JD, Faro S, Force RW, Foxman B, Ledger WJ (1998). Vulvovaginal candidiasis: epidemiologic, diagnostic, and therapeutic considerations. Am J Obstet Gynecol.

[3] Achkar JM, Fries BC (2010). *Candida* infections of the genitourinary tract. Clin Microbiol Rev.

[4] Peters BM, Yano J, Noverr MC, Fidel PL (2014). *Candida vaginitis*: when opportunism knocks, the host responds. PLoS Pathog.

[5] Vander H, Prabha V (2015). Evaluation of fertility outcome as a consequence of intravaginal inoculation with sperm-impairing micro-organisms in a mouse model. J Med Microbiol.

[6] Mosca V, Arita GS, Sakita KM, Rodrigues-Vendramini FAV, Faria DR (2025). *Candida albicans* migrates itself from the vagina to the uterus and ovaries in estrogenized mice. Braz J Microbiol.

[7] Disha T, Haque F (2022). Prevalence and risk factors of vulvovaginal candidosis during pregnancy: a review. Infect Dis Obstet Gynecol.

[8] P Córdova AL, Z M Fontanella S, Colonetti T, Rodrigues Uggioni ML, Grande AJ (2024). Role of vulvovaginal candidiasis infection in infertility: systematic review and meta-analysis. Braz J Microbiol.

[9] Tuttle JP, Bannister ER, Derrick FC (1977). Interference of human spermatozoal motility and spermatozoal agglutination by *Candida albicans*. J Urol.

[10] Golshani M, Taheri S, Eslami G, Suleimani Rahbar AA, Fallah F (2006). Genital tract infection in asymptomatic infertile men and its effect on semen quality. Iran J Public Health.

[11] Burrello N, Salmeri M, Perdichizzi A, Bellanca S, Pettinato G (2009). *Candida albicans* experimental infection: effects on human sperm motility, mitochondrial membrane potential and apoptosis. Reprod Biomed Online.

[12] Sasikumar S, Dakshayani D, Sarasa D (2013). An investigation of DNA fragmentation and morphological changes caused by bacteria and fungi in human spermatozoa. Int J Curr Microbiol App Sci.

[13] Sasikumar S, Dakshayani D, Franklin A, Rajkumar S (2013). An in-vitro study of effectiveness of uropathogenic yeasts on male infertility. Int J Curr Microbiol App Sci.

[14] Castrillon-Duque EX, Puerta Suarez J, Cardona Maya WD (2018). Yeast and fertility: effects of in vitro activity of *Candida* spp. on sperm quality. J Reprod Infertil.

[15] Leitich H, Kiss H (2007). Asymptomatic bacterial vaginosis and intermediate flora as risk factors for adverse pregnancy outcome. Best Pract Res Clin Obstet Gynaecol.

[16] Martin DH (2012). The microbiota of the vagina and its influence on women’s health and disease. Am J Med Sci.

[17] Mosca V, S Arita G, Vilegas LV, Faria DR, Sakita KM (2020). Vulvovaginal candidiasis in a murine model of diabetes emphasizing the invasive ability of etiological agents. Future Microbiol.

[18] Bonfim AP, Sakita KM, Faria DR, Arita GS, Vendramini FAVR (2020). Preclinical approaches in vulvovaginal candidiasis treatment with mucoadhesive thermoresponsive systems containing propolis. PLoS One.

[19] Bonfim AP, Sakita KM, Faria DR, Arita GS, Rodrigues-Vendramini FA (2023). Successful treatment of experimental murine vulvovaginal candidiasis with gentian violet. Future Microbiol.

[20] Naglik JR, Fidel PL, Odds FC (2008). Animal models of mucosal *Candida* infection. FEMS Microbiol Lett.

[21] Fidel PL, Barousse M, Espinosa T, Ficarra M, Sturtevant J (2004). An intravaginal live *Candida* challenge in humans leads to new hypotheses for the immunopathogenesis of vulvovaginal candidiasis. Infect Immun.

[22] Meizoso T, Rivera T, Fernández-Aceñero MJ, Mestre MJ, Garrido M (2008). Intrauterine candidiasis: report of four cases. Arch Gynecol Obstet.

[23] Georgescu TA, Lisievici AC, Munteanu O, Furtunescu FL, Bratu OG (2020). Congenital systemic candidiasis: a comprehensive literature review and meta-analysis of 44 cases. Rom J Morphol Embryol.

[24] Teacoe DA, Cormoș RC, Toma DA, Ștef L, Cucerea M (2024). Congenital sepsis with *Candida albicans*-A rare event in the neonatal period: report of two cases and literature review. Microorganisms.

[25] Mosci P, Pietrella D, Ricci G, Pandey N, Monari C (2013). Mouse strain-dependent differences in estrogen sensitivity during vaginal candidiasis. Mycopathologia.

[26] Zhao X, Sun D, Zhang A, Huang H, Li Y (2023). *Candida albicans*-induced activation of the TGF-β/Smad pathway and upregulation of IL-6 may contribute to intrauterine adhesion. Sci Rep.

[27] Ali SR, Rasheed KN, Hameed AK (2021). Experimental infection effect with *Candida albicans* in female rats’ uterus. Nat Volatiles Essent Oils.

[28] Chen Z, Kong X (2007). Study of *Candida albicans* vaginitis model in Kunming mice. *J Huazhong Univ Sci Technolog Med Sci*.

